# Male Nursing

**Published:** 1920-07-31

**Authors:** H. W. G. Brown

**Affiliations:** Late S.-Sgt. Dispenser. C.A.M.C.


*To the Editor of* The Hospital.

### Male Nursing.

*To the Editor of* The Hospital.

Sir,—Permit me, through your valuable columns, to call particular attention to the necessity for male nursing and training, which are as essential as they are for women. During the war it was my duty to a great extent to find and train soldiers for nursing duties. In the Army our Services in Medical Corps detail were sadly lacking, and consequently, where such duties had to be carried out by a male staff, great delay, hindrance, and—shall I say?—neglect followed, owing to the fact that proper care and nursing were unobtainable. In our special hospitals this was daily experienced. Accordingly, as in other departments, the male nursing staffs really did not begin to know their worth until the final shot was fired and peace declared. To-day, as when at war, the civilian population demand the services of properly trained male nurses. Why could not dispensers and masseurs, say, qualify under a system of training, and, in addition to the above qualifications and duties, take or get the privilege of taking a two-years' training in hospital routine, administration, and qualification? As a dispenser I have found such training and experience a valuable asset to my calling when serving, especially in C.A.M.C. In visiting other hospitals I have noticed the shortage of skilled male help, which had to be carried out accordingly by a female staff.—Yours truly,

H. W. G. Brown,
Late S.-Sgt. Dispenser, C.A.M.C.

